# Transcriptome profiling and weighted gene co-expression network analysis reveal changes of hub genes and molecular pathways in rat lungs following deep hypothermic circulatory arrest

**DOI:** 10.1371/journal.pone.0328887

**Published:** 2025-08-14

**Authors:** Lei Wang, Qingtong Wu, Yuzuo Lin, Ziyan Lin, Guodong Zhong, Liangwan Chen

**Affiliations:** 1 Department of Cardiovascular Surgery, Fujian Medical University Union Hospital, Fuzhou, China; 2 Key Laboratory of Cardio-Thoracic Surgery (Fujian Medical University), Fujian Province University, Fuzhou, China; 3 Union College of Clinical Medicine, Fujian Medical University, Fuzhou, China; 4 Department of Pathology, Fujian Province Second People’s Hospital: The Second Affiliated Hospital of Fujian University of Traditional Chinese Medicine, Fuzhou, China; 5 Engineering Research Center of Tissue and Organ Regeneration, Fujian Province University, Fuzhou, China; Royal Holloway University of London, UNITED KINGDOM OF GREAT BRITAIN AND NORTHERN IRELAND

## Abstract

**Background:**

The incidence of acute lung injury (ALI) following aortic dissection repair surgery that involves deep hypothermic circulatory arrest (DHCA) is notably high. We analyzed hub genes and signaling pathways in rat lung tissues post-DHCA using transcriptome sequencing and weighted gene co-expression network analysis (WGCNA).

**Methods:**

A rat model of DHCA was established, and lung tissues were collected after the procedure. High-throughput transcriptome sequencing was employed to assess gene expression differences between the DHCA group and the non-DHCA group. The DESeq2 method was utilized to analyze differentially expressed genes (DEGs) between these two groups, with further screening for hub genes and their upstream molecules conducted using WGCNA, protein-protein interaction (PPI) networks, and the iRegulon plugin. Biological functions of hub genes were examined via Gene Ontology and Kyoto Encyclopedia of Genes and Genomes analyses. The changes in mRNA and protein levels of hub genes across both groups were evaluated through experimental verification.

**Results:**

A total of 438 DEGs were identified when comparing the DHCA group to the control group. WGCNA further revealed 197 key genes. Subsequent PPI analysis led to the identification of eight hub genes: FOS, FOSB, JUN, EGR1, ATF3, NR4A1, CCN1, and ZFP36. The hub genes were primarily associated with inflammation, cell apoptosis, and cellular immune responses. ATF3 and SRF may serve as potential upstream regulators. The experimental findings further corroborated that substantial alterations took place in these hub genes, accompanied by significant injury of lung tissue during DHCA.

**Conclusion:**

DHCA significantly altered gene expression patterns in rat lung tissues. The identified hub genes and signaling pathways related to inflammation and apoptosis may serve as potential therapeutic targets for lung injury following DHCA.

## 1. Introduction

Deep hypothermia circulatory arrest (DHCA) is a specialized cardiopulmonary bypass (CPB) technique primarily utilized in surgical procedures for aortic dissection (AD) involving the aortic arch, as well as in complex congenital heart diseases. During DHCA, systemic circulation is suspended at deep hypothermic temperature (<20°C), providing surgeons with bloodless operative fields that facilitate seamless surgical interventions. Concurrently, the DHCA technique significantly reduces overall metabolic rates, thereby preserving organ function. The implementation of regional cerebral perfusion technique has mitigated neurological complications following DHCA; however, other organ dysfunction, such as lung injury post-DHCA, remain unresolved. Aortic surgery itself constitutes an independent risk factor for postoperative acute respiratory distress syndrome (ARDS) [1]. Postoperative hypoxemia and acute lung injury (ALI), or ARDS occur with increased frequency after DHCA, with the incidence of respiratory failure reaching as high as 13.3% [2]. Pathological changes associated with lung injury following DHCA can be characterized by pulmonary edema, atelectasis, increased intrapulmonary shunting, impaired gas exchange, and decreased pulmonary oxygenation function. These alterations may be attributed to several factors: (1) The interaction of blood components with the non-physiological surfaces of the CPB circuit induces systemic inflammation. This process activates a significant number of neutrophils within the pulmonary circulation, prompting them to secrete various inflammatory mediators. Consequently, this damages the basement membrane of both pulmonary capillary endothelium and alveolar epithelium, resulting in increased permeability and adversely affecting the functionality of pulmonary surfactant. Such changes ultimately lead to pulmonary edema and atelectasis [3]. (2) DHCA compromises vascular-dependent diastolic function through reduced production of nitric oxide (NO) and acetylcholine by endothelial cells while simultaneously increasing levels of endothelin-1 and its receptors in lung endothelial cells [4]. This disruption alters the balance between vasodilation and vasoconstriction, contributing to postoperative pulmonary hypertension. (3) The non-pulsatile blood flow that occurs after aortic cross-clamping during CPB—alongside cessation of blood flow in the pulmonary arteries and diminished bronchial arterial supply—results in inadequate perfusion during DHCA to meet metabolic demands. This insufficiency can precipitate pulmonary ischemia; subsequent reperfusion at later stages may further exacerbate lung injury. (4) The cessation of mechanical ventilation during DHCA can result in pulmonary atelectasis and intrapulmonary shunting. (5) Deep hypothermia activates platelets and coagulation factors, potentially leading to increased bleeding and the need for transfusions, which may cause transfusion-related ALI [5]. The aforementioned factors are primarily associated with an inflammatory response, an imbalance in pulmonary vasodilation, pulmonary ischemia/reperfusion injury, and inadequate pulmonary ventilation/perfusion. These elements may contribute to lung injury following DHCA. However, research on the specific molecular mechanisms and biomarkers related to lung injury after DHCA remains limited. Patients experiencing postoperative lung injury following DHCA can result in ARDS, refractory hypoxemia, significantly prolonged mechanical ventilation duration, extended ICU stays, and longer overall hospitalizations [6]. Delayed extubation heightens the risk of infection, limits patient mobility, may lead to secondary deep vein thrombosis. Furthermore, hypoxia adversely affects the function of other organs, potentially triggering multiple organ dysfunction syndrome, increasing the risk of patient mortality [6]. Besides, lung injury escalates the need for bronchoscopic interventions, advanced antibiotic therapies or extracorporeal membrane oxygenation support, consequently placing an additional burden on medical resources [7]. Additionally, lung injury may contribute to pulmonary fibrosis and chronic restrictive lung disease while diminishing exercise tolerance [8], ultimately impacting long-term quality of life. Consequently, it is imperative to investigate the specific molecular mechanisms underlying lung injury post-DHCA.

Microarray analysis and bioinformatics have progressed significantly in recent years. Array analysis facilitates a systematic and comprehensive comparison of expression profiles between diseased and normal tissues, while bioinformatics elucidates significant molecular networks from whole-genome data, thereby revealing new disease mechanisms and potential biomarkers for clinical application. Consequently, bioinformatics is increasingly utilized in studies of cardiovascular diseases, which may also provide insights into the underlying pathogenesis of ALI post-DHCA. A study has analyzed lung metabolomics following DHCA in young pigs, demonstrating that DHCA is linked to abnormal lung metabolism. Specifically, DHCA disrupts metabolic pathways involving amino acids, carbohydrates, lipids, steroids, vitamins, as well as the oxidation-reduction pathway [9]. However, transcriptome sequencing results for lungs post-DHCA have yet to be reported; thus far, research on the mechanisms underlying ALI post-DHCA remains limited. In this study, we constructed a rat model of DHCA to investigate the transcriptomic response of lung tissues under these conditions. This approach effectively mitigated the influence of surgical factors on outcomes and further clarified the possible mechanisms of ALI post-DHCA.

## 2. Materials and methods

### 2.1 Construction of a rat DHCA model

Twelve specific pathogen free male SD rats, each weighing between 400g and 450g and aged 10 weeks, were procured and allowed free access to food in an environment maintained at a temperature range of 18–26 °C, with humidity levels between 40–70%, and a normal day-night cycle of 12 hours. The experiment commenced after one week of acclimatization. The rats were randomly assigned into two groups: the DHCA group (n = 6) and the non-DHCA control group (n = 6). Following a fasting period of six hours prior to surgery, anesthesia was induced using sevoflurane. The limbs of the rats were secured onto the operating table, followed by intubation via an oral tube with a 14G anesthesia trocar; mechanical ventilation was subsequently initiated. The initial respiratory rate was set at 70 breaths per minute, with tidal volumes ranging from 8–10 ml/kg. Anesthesia maintenance involved inhaling a mixture of 100% pure oxygen supplemented with 3% sevoflurane. Mechanical ventilation was temporarily halted during CPB but resumed following CPB weaning. Ventilator parameters were adjusted based on arterial blood gas values throughout the procedure.

Rectal temperature was monitored using an electronic thermometer, which was lubricated with paraffin oil and inserted 5 cm into the anus. A multistage venous drain tube (4.5 mm in diameter, 50 cm in length) was inserted via the right external jugular vein into both the inferior vena cava and right atrium to function as a venous outflow line, and systemic heparinization was achieved by administering 200 IU of heparin following the establishment of the cannulae. A 20G cannula was employed for caudal artery cannulation to serve as a arterial perfusion line. Mean arterial blood pressure was monitored through a 22G cannula needle inserted into the left femoral artery. All cannulated vessels were ligated distally using silk threads; the threads also secured the proximal end of each cannula to prevent displacement. The CPB circuit comprised an external jugular vein cannula, a venous reservoir, a roller pump (Stockert III, Germany), a heat exchanger (Xi Jing, Xi’an, China), a temperature-regulating water bank (Stockert III, Germany), an oxygenator (Xi Jing, Xi’an, China), and an arterial circuit. The oxygenator consisted of two acrylic sheets enclosing a disposable three-layer hollow fiber membrane. Prior to use, the CPB circuit was primed and deflated with 10 ml multiple electrolytes injection (Jiameina, China) and 5 ml 6% hydroxyethyl starch (HES) 130/0.4 (Volulyte, China).

**Fig 1:**
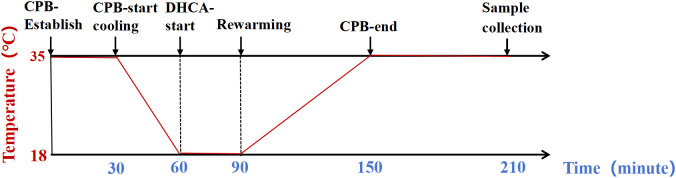
The experimental process of a rat DHCA model. CPB: cardiopulmonary bypass; DHCA: deep hypothermic circulatory arrest.

After establishing the cannulas, they were connected to the CPB circuit and equipment, followed by the initiation of CPB. The initial CPB flow rate was set between 120–160 ml/kg/min, which is comparable to the physiological cardiac output observed in rats. This flow rate was gradually adjusted—either reduced or increased by one half to one third—during both cooling and rewarming phases of CPB. To ensure adequate organ perfusion, mean arterial pressure was maintained above 40 mmHg. Following a maintenance period of normothermia for three minutes at the onset of CPB, whole-body cooling commenced utilizing a heat exchanger and temperature-regulated water tank. Concurrently, local brain cooling was achieved through the application of ice packs. These measures collectively facilitated a reduction in rectal temperature to 18°C within a span of 30 minutes based on prior research experience [10]. At this point, both the roller pump for CPB and ventilator support were halted to maintain all organs in a state of no perfusion. After sustaining DHCA for 30 minutes, CPB was reinstated while slowly rewarming the rats over approximately one hour until rectal temperature reached 35°C; subsequently, CPB was discontinued. The total duration of CPB amounted to roughly 150 minutes. The ventilator was maintained for one hour following the CPB weaning. The lungs were excised and collected for further analysis. Some tissue samples were promptly snap-frozen in liquid nitrogen. The remaining portions of the tissues were immersed in 4% paraformaldehyde for subsequent pathological examination. Subsequently, the rats were over-anesthetized and euthanized using sodium pentobarbital at a dosage of 100 mg/kg. In contrast, the rats in the sham-operated group underwent only anesthesia induction, intubation, and systemic heparinization for approximately 210 minutes before samples collection using identical procedures as previously described. The construction of the DHCA model is illustrated in Fig 1 and S1 Fig. All experiments adhered to internationally recognized animal ethical standards and received approval from the Animal Ethics Committee of Fujian Medical University (IACUC FJMU 2024-Y-1767).

### 2.2 Collection of lung samples, extraction of total RNA, and construction of mRNA libraries

Rat lungs were promptly harvested following the DHCA model. Total RNA was isolated from 60 mg of lung tissue using Trizol reagent (Invitrogen, Carlsbad, CA, USA). The quality and quantity of the total RNA were assessed with a NanoDrop spectrophotometer and an Agilent 2100 Bioanalyzer (Thermo Fisher Scientific, MA, USA). Each sample was confirmed to meet the quality standards with a 260/280 ratio exceeding 2.0 and an RNA integrity number (RIN) greater than 7. Subsequently, cDNA libraries were constructed from 1 mg of total RNA and sequenced on an Illumina NovaSeq 6000 platform (Tsingke-Beijing, China) utilizing the Illumina TruSeq RNA sample preparation kit.

### 2.3 Transcriptome sequencing and data analysis

The quality of the raw sequencing data was assessed using FastQC, followed by quality filtering and splicing with Trimmomatic. High-quality sequence alignment to the rat reference genome was performed utilizing Hisat2 software (Version ensemble107). Gene counts were obtained using StringTie. Differential expression genes (DEGs) were identified through DESeq2 method, applying screening criteria of adjusted *P*-value < 0.05 and |log2FC| ≥ 1. Heatmaps and volcano plots for DEGs were generated using the “pheatmap”, “ggVolcano” and “ggplot2” R packages.

### 2.4 Weighted gene co-expression network analysis (WGCNA)

WGCNA was implemented to systematically characterize co-expression patterns and identify biologically meaningful network structures in gene expression data. The workflow proceeded as follows: First, genes with a standard deviation exceeding 0.5 across samples were retained to ensure expression variability. All samples were then subjected to clustering analysis, and outliers exhibiting abnormal clustering patterns were excluded to enhance data robustness. Next, a scale-free co-expression network was subsequently constructed using a one-step network construction algorithm. The soft thresholding power (β = 1) was optimized through the “pickSoftThreshold” R function to maximize network connectivity while preserving scale-free topology properties. Following this, the adjacency matrix derived from pairwise gene correlations was converted into a topological overlap matrix (TOM) to quantify network interconnectedness, with the corresponding dissimilarity measure (1-TOM) calculated for downstream analysis. Hierarchical clustering coupled with dynamic tree-cutting algorithms was applied to delineate co-expression modules, with a minimum module size threshold set to 60 genes. Gene significance (correlation with clinical traits) and module membership (intramodular connectivity) were quantified to identify clinically relevant modules. Finally, the co-expression network architecture was visualized using heatmap or equivalent tools. All computational procedures were executed using custom R scripts, which were provided in the S1 File.

### 2.5 Functional enrichment analysis

The modular genes obtained from WGCNA were candidate key genes for alteration in the lungs after DHCA. Subsequently, Gene Ontology (GO) functional analysis and Kyoto Encyclopedia of Genes and Genomes (KEGG) pathway analysis were conducted on these 197 candidate hub genes utilizing the “clusterProfiler” R package. The GO analysis encompassed biological processes (BP), cellular components (CC), and molecular functions (MF). A significance threshold for enrichment was set at adjusted *P* < 0.05.

### 2.6 Protein-protein interaction (PPI) network construction and hub gene identification

The PPI network of the candidate hub genes was constructed utilizing the STRING database (http://string-db.org). A combined score of 0.4 was set as the threshold to consider edges between two proteins as signiﬁcant. Subsequently, this PPI network was visualized using Cytoscape software. The CytoHubba plugin for Cytoscape was employed to identify the most significant interacting genes utilizing the Maximal Clique Centrality (MCC) algorithm from the CytoHubba plugin. The greater the MCC score of a gene, the more pronounced its centrality and functional significance within the network. The eight genes with the highest MCC scores were identified as hub genes. Then, the differences in mRNA expression levels of these hub genes between the DHCA group and the control group in the sequencing data were compared. Besides, GO and KEGG analyses were performed again on these eight hub genes. Furthermore, the predictive efficacy of the hub genes for lung gene alterations following DHCA was evaluated using receiver operating characteristic (ROC) curves. A hub gene with an area under the ROC curve (AUC) of ≥ 0.7 was deemed clinically valuable.

### 2.7 Identification of upstream regulators of hub genes

The iRegulon plugin (v1.3) within the Cytoscape tool was employed to identify upstream transcription factors (TFs) governing the regulation of hub genes [11]. This study integrated 9713 position weight matrices (PWMs) and 1120 raw signal tracks from ENCODE ChIP-seq data, with a focus on the 20-kilobase regions both upstream and downstream of transcription start sites (TSS). The analysis was conducted under stringent thresholds to ensure specificity: normalized enrichment score (NEScore) ≥ 3, maximum false discovery rate (FDR) for motif similarity ≤ 0.001, and an AUC threshold set at 0.03. By cross-validating motif enrichment against ChIP-seq data, we identified significantly regulated TFs along with their target genes, followed by an analysis of their functional associations.

### 2.8 Experimental verification

For real-time polymerase chain reaction (RT-PCR), the total mRNA was extracted using the RNeasy Mini Kit (Qiagen, USA), followed by reverse transcription with the PrimeScript™RT Master Mix Kit (Takara, Japan). RT-PCR amplification was then performed using TB Green® Premix Ex Taq™ II (Takara, Japan). β-actin served as the reference gene. The primer sequences for eight rat hub genes were provided in S1 Table. All PCR samples were quantified utilizing the comparative CT method to determine relative mRNA levels.

Hematoxylin and eosin (HE) staining. After preparing paraffin-embedded sections, the slides were deparaffinized using xylene, followed by rehydration through a series of graded alcohols ranging from 100% to 70%. Stain the nuclei with hematoxylin for 8 minutes, then rinse in tap water to achieve “blueing”. Counterstain the cytoplasm with eosin for 35 seconds, followed by a brief rinse. Proceed with dehydration using alcohols and clearing with xylene, after which mount the slides using resin. Finally, examine the sections using a microscope.

Immunohistochemistry (IHC) staining. Deparaffinize and rehydrate the slides according to the procedures outlined in the preceding steps. Antigen retrieval was performed using a citrate buffer (0.01M, pH 6.0) with boiling for 15 minutes. Endogenous peroxidase activity was blocked by incubating the slides in 3% hydrogen peroxide for 30 minutes. Subsequently, a blocking buffer containing normal goat serum (C-0005) was applied at 37 °C for 20 minutes. Primary antibodies were then incubated overnight at 4 °C: FOS (Bioss, bs-0469R), FOSB (Bioss, bsm-52071R), Jun (Bioss, bs-0670R), EGR1 (Bioss, bs-1076R), ATF3 (Bioss, bs-0519R), NR4A1 (Bioss, bsm-52972R), CCN1 (Bioss, bs-1290R) and ZFP36 (Bioss, bs-20893R). This was followed by conjugation with an HRP-conjugated secondary antibody from CST (#7074) and DAB staining from Ventana, USA. The immunohistochemistry scores were categorized into four distinct levels: negative, weakly positive, moderately positive, and strongly positive.

### 2.9 Statistical analyses

Statistical analyses were conducted using R software (Version 4.3.3) and GraphPad Prism software (Version 9.5.0). Comparisons between groups were performed utilizing Student’s *t*-test. *P* < 0.05 was deemed statistically significant.

## 3. Results

### 3.1 Model construction

The modeling process of DHCA in rats was conducted with relative ease, maintaining hematocrit levels at an appropriate 20% throughout the procedure, which is regarded as a suitable level [12]. Concurrently, we collected kidney, brain, and liver tissue samples from the rats for sequencing analysis. To guarantee the acquisition of high-quality samples for transcriptome sequencing, we ultimately adhered to the minimum sample requirements: three cases each from both the DHCA and control groups were selected for transcriptome analysis [13]. In order to minimize potential omissions of DEGs between the two groups, we increased both the volume and depth of sequencing for individual samples to compensate for any limitations posed by sample size. Furthermore, we will conduct additional experimental validation on key DEGs identified through this analysis.

### 3.2 Identification of DEGs

**Fig 2:**
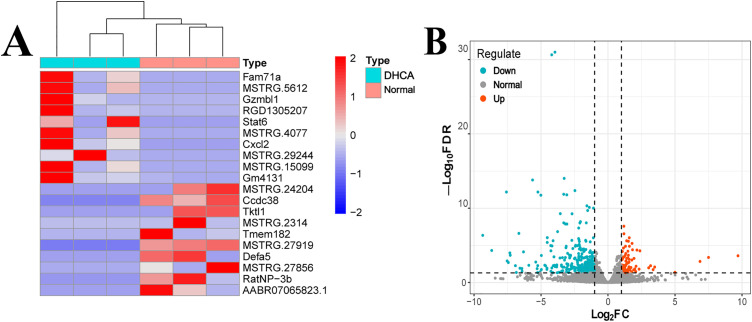
Heatmap and volcano plot of DEGs between the DHCA group and the control group. **(A)** Heatmap of DEGs identified using the DESeq2 method between the DHCA group and the control group. **(B)** Volcano plot of DEGs identified using the DESeq2 method between the DHCA group and the control group.

In this study, lung tissues were collected for whole transcriptome sequencing at 1 hour after CPB weaning in order to compare the changes in gene expression between DHCA and non-DHCA control groups (n = 3 per group). Of the 21,474 transcripts detected in the sequencing, 19,684 transcripts were successfully mapped to the rat genome annotation. A total of 438 DEGs were identified using the DESeq2 method, of which 101 were up-regulated and 337 were down-regulated (Fig 2, S2 Table.)

### 3.3 WGCNA construction

**Fig 3:**
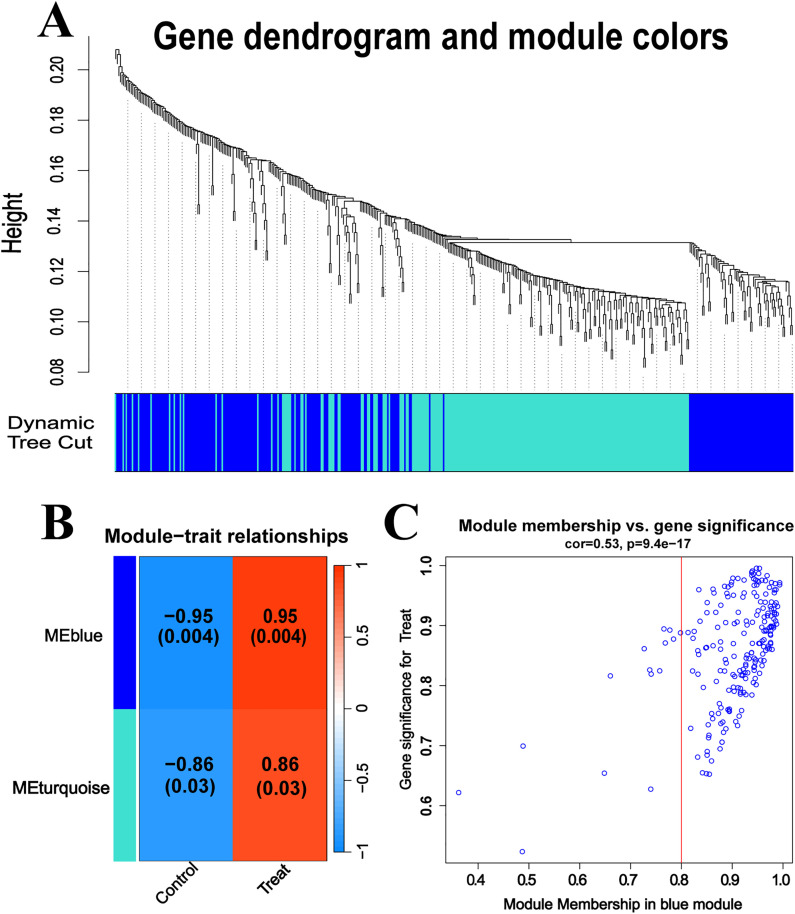
WGCNA and identification of significant modules. **(A)** Dendrogram illustrating the clustering of 438 genes based on the topological overlap matrix (1-TOM). **(B)** Module-trait heatmap displaying clustered gene modules along with their correlation to genetic alteration in lungs following DHCA, including corresponding correlation coefficients and *P* values for each module. **(C)** Scatter plot indicating a significant positive correlation between the blue module and genetic alteration in lungs following DHCA.

WGCNA analysis was conducted on the 438 DEGs to evaluate gene expression in lung tissues following DHCA (Fig 3A). We identified two distinct functional modules (Fig 3B). Through positive correlation coefficient analysis, we determined a significant correlation between the blue module, which comprises 197 key genetic alterations in lungs following DHCA (correlation coefficient = 0.95, *P* = 0.004, Fig 3C; S3 Table.).

### 3.4 Functional enrichment analysis

**Fig 4:**
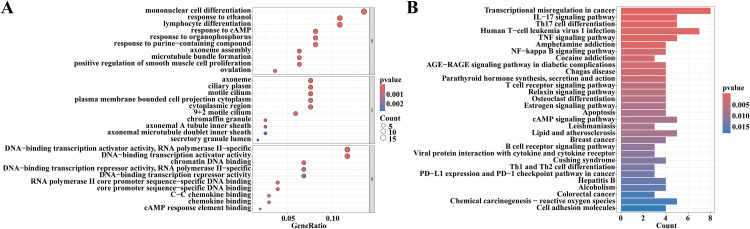
Functional enrichment analysis of candidate hub genes. **(A)** GO enrichment analysis of the candidate hub genes. **(B)** KEGG pathway analysis of the candidate hub genes.

The 197 key genes identified through the WGCNA method, which were associated with altered genes in lungs following DHCA, were recognized as candidate key genes that may play a critical role in changes in lung function post-DHCA. Subsequently, these genes underwent GO and KEGG analyses to explore their potential biological functions (Fig 4A and 4B). GO analysis (Fig 4A) revealed that, concerning BP, the candidate key genes were primarily linked to monocyte and lymphocyte differentiation, responses to cyclic adenosine monophosphate (cAMP), and reactions to external stimuli. In terms of CC, they were predominantly associated with the cytoplasm, plasma membrane, and intracellular regions of ciliated cells. Regarding MF, they were mainly related to DNA-binding transcription activator activity, RNA polymerase II-specific; DNA-binding transcription activator activity; C-C chemokine binding; chemokine binding, and other molecular functions. KEGG analysis indicated that the candidate key genes were chiefly connected to signaling pathways involving IL-17, TNF, NF-κB, cAMP, reactive oxygen species, T cell receptor signaling pathways as well as B cell receptor signaling pathways; additionally involved in Th17 differentiation along with Th1 and Th2 cell differentiation and apoptosis processes (Fig 4B).

### 3.5 PPI network construction and hub gene screening

**Fig 5:**
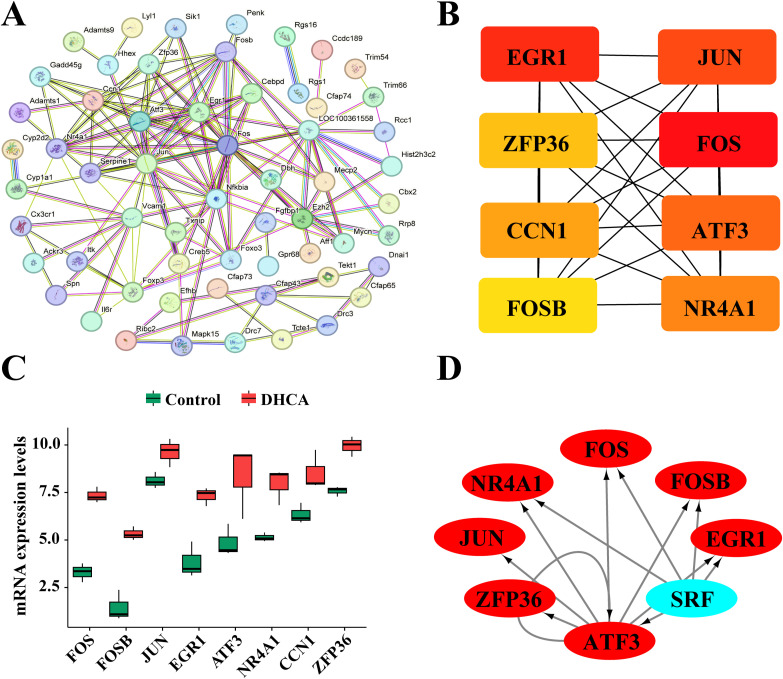
Construction of PPI network and screening of hub genes. **(A)** PPI network illustrating candidate hub genes. **(B)** The top eight hub genes identified utilizing the MCC algorithm from the CytoHubba plugin. **(C)** Differential mRNA expression levels of the top 8 hub genes between the DHCA group and control group in the sequencing data. **(D)** The transcription factors of hub genes predicted by iRegulon in Cytoscape. PPI: protein-protein interaction. MCC: Maximal Clique Centrality.

A PPI network comprising 197 genes identified through WGCNA analysis was constructed using the String online tool (Fig 5A). The top eight genes within the connected nodes were visualized utilizing the CytoHubba plugin in Cytoscape software (Fig 5B). These hub genes included FOS, FOSB, JUN, early growth response 1 (EGR1), activating transcription factor 3 (ATF3), neurotensin receptor 4 alpha 1 (NR4A1), cellular communication network factor 1 (CCN1), and zinc finger protein 36 (ZFP36). A darker coloration of a gene indicated a higher MCC score, suggesting its more significant role played in potential changes of lung function following DHCA. Notably, the FOS gene exhibited the darkest hue with the highest MCC score among these candidates. The MCC scores for these eight hub genes were presented in S4 Table. The differences in mRNA levels of these hub genes between the DHCA group and control group in the sequencing data were illustrated in Fig 5C. The functional enrichment analysis of these eight hub genes, encompassing both GO and KEGG, were presented in S2 Fig. The GO analysis revealed that these genes were primarily associated with the cellular response to cAMP, responses to polypeptide hormone stimulation, positive regulation of microRNA transcription, cellular responses to hypoxia, as well as RNA and DNA synthesis. The KEGG analysis indicated that these genes were predominantly linked to signaling pathways such as IL-17, MAPK, GnRH, TLR, TNF, apoptosis, along with Th1, Th2, and Th17 cell differentiation processes. The AUC values for all these hub genes consistently reached 1.000, as shown in S3 Fig, indicating that all eight genes demonstrate potential for enhanced predictive performance regarding lung gene alterations following DHCA.

### 3.6 Identification of upstream regulators of hub genes

A gene regulatory network was established, consisting of eight genes, among which two were identified as upstream regulators: ATF3 and SRF. These genes exhibited the highest NEScores of 6.437 and 6.374, respectively. ATF3 regulates seven hub genes, including FOS, FOSB, JUN, EGR1, NR4A1, and ZFP36. In contrast, SRF regulates five hub genes: FOS, FOSB, EGR1, NR4A1, and ATF3 (Fig 5D).

### 3.6 Comparison of mRNA levels of eight hub genes in rat lung tissues between the control and the DHCA groups

**Fig 6:**
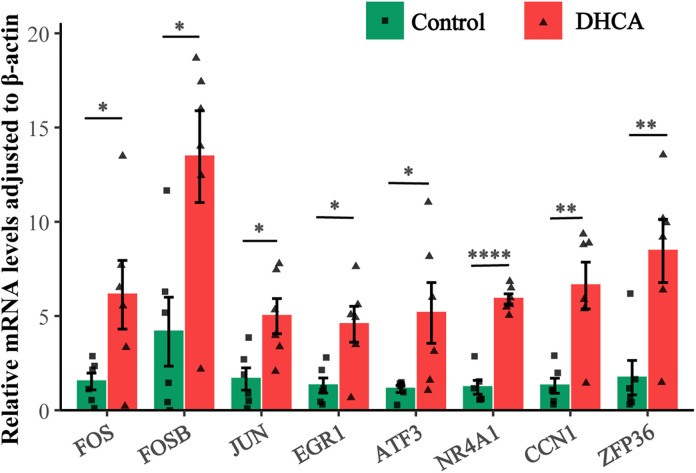
Comparison of mRNA levels of hub genes between the DHCA and the control groups. *P < 0.05, **P < 0.01, ****P < 0.0001.

The RT-PCR results indicated that the mRNA levels of eight hub genes in the DHCA group were significantly higher than those in the control group (Fig 6), verifying the significant upregulation of these hub genes in lung tissues following DHCA.

### 3.7 Comparison of HE staining and IHC staining of hub genes in lung tissues between the control and the DHCA groups

**Fig 7:**
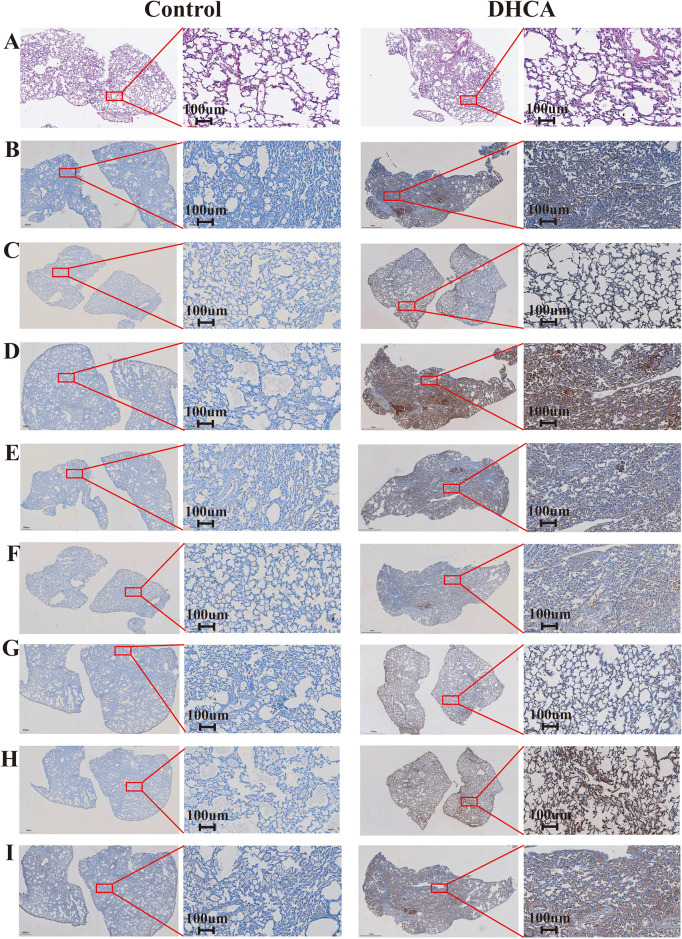
Comparison of HE staining and IHC staining of hub genes in lung tissues between the DHCA and the control groups. **(A)** The results of HE staining in lung tissues between the two groups. (B-I) The analysis of IHC staining for hub genes in lung tissues was conducted between the two groups. The genes examined, in sequence, included FOS, FOSB, JUN, EGR1, ATF3, NR4A1, CCN1, and ZFP36. HE: hematoxylin and eosin; IHC: immunohistochemistry.

The results of HE staining (Fig 7A) revealed that, in comparison to the control group, pulmonary edema and pulmonary alveolar septal hemorrhage were significantly more pronounced in the DHCA group. Additionally, necrosis and shedding of alveolar epithelial cells were observed. These findings indicated a more severe lung injury in the DHCA group. Moreover, these findings further supported the notion that the identified hub genes may be associated with the activation of pathways such as apoptosis. Besides, the IHC staining analysis (Fig 7B-7I) demonstrated that the protein expression levels of all eight hub genes were significantly elevated in the DHCA group when compared to those in the control group. The DHCA group exhibited a strong positive response, whereas the control group demonstrated either a negative or weakly positive reaction.

## 4. Discussion

In this study, we identified eight hub genes altered in rat lung tissues following DHCA. These genes—FOS, FOSB, JUN, EGR1, AFT3, NR4A1, CCN1, and ZFP36—were revealed through transcriptome sequencing and DEGs, complemented by a WGCNA and PPI methods. Furthermore, our findings indicated an upregulation of pathways associated with inflammation, oxidative stress, cell apoptosis, as well as cellular immune responses in the lung tissues post-DHCA. Besides, our pathological experiments indicated that lung tissue lesions were significantly more severe following DHCA than in the control group. This study addresses a critical gap in understanding the transcriptomic changes and potential molecular pathways affecting the lungs after DHCA. It provided valuable insights into the molecular mechanisms underlying the impact of DHCA on lung tissues.

Eight DEGs that were significantly elevated in lung tissues following DHCA primarily function as early immediate-response genes to stress. Various stimuli can induce the upregulation of these gene expressions, which are involved in cellular proliferation, differentiation, apoptosis, inflammation, and immune responses [14–18]. Our study demonstrated that the FOS, FOSB, and JUN genes exhibited significant upregulation in the DHCA group compared to the non-DHCA group. FOS, FOSB, and JUN serve as major subunits of activator protein-1 (AP-1) in mammals. AP-1 is a transcription factor that responds rapidly to stressors. Most studies indicate that AP-1 acts as a pro-injury factor by regulating cell proliferation, apoptosis, and the expression of inflammation-related genes [14]. Notably, increased expression of FOS protein in lung tissues within a rat model of ventilator-associated lung injury exacerbates lung inflammation and damage; conversely, inhibitors targeting FOS/AP-1 have been shown to protect lung function [19]. The expression levels of NF-κB and FOSB were found to be up-regulated in the lung tissues of a rat model associated with shockwave-induced lung injury, which subsequently promoted the release of inflammatory factors such as IL-6 and IL-8, along with oxidative stress, ultimately leading to lung damage [20]. In hub genes, FOS exhibited the most significant upregulation in lung tissue following DHCA. The increased expression of FOS, FOSB, and JUN may contribute to inflammation and oxidative stress while also influencing the cell cycle of lung epithelial cells, ultimately leading to lung injury.

EGR1 is an intranuclear transcription factor that belongs to the EGR family of Cys2His2-type zinc finger proteins. It can be activated by various stimuli, including growth factors, hypoxia, H_2_O_2_, ultraviolet light, and inflammatory mediators [16]. Most studies indicated that EGR1 exerts a detrimental effect. The EGR1-associated pro-inflammatory axis plays a crucial role in IgG immune complex-induced ALI [21]. Furthermore, EGR-1 has been shown to promote lipopolysaccharide (LPS)-induced ALI; conversely, inhibition of EGR-1 expression offers protective effects against ALI [22]. Compounds found in cigarette smoke can induce EGR1 expression through airway hyperresponsiveness (AHR), which subsequently leads to bronchial epithelial cell apoptosis and chronic obstructive pulmonary disease (COPD) [23]. Additionally, the EGR1-related pathway in endothelial cells derived from pluripotent stem cells contributes to the development of pulmonary hypertension associated with Down syndrome [24]. Therefore, it can be inferred that EGR1 may play a significant role in contributing to lung injury following DHCA, primarily by promoting pulmonary inflammatory responses, facilitating cell apoptosis, and exacerbating pulmonary hypertension.

ATF3 is recognized as an early immediate-response gene associated with apoptosis, immunity, and metabolism [15]. Some research has indicated that ATF3 may promote cell death and contribute to tissue injury. For example, Circexoc5 enhances ferroptosis and exacerbates sepsis-induced ALI via the IGF2 BP2/ATF3 signaling pathway [25]. Arsenic induces apoptosis in human bronchial epithelial cells by rapidly elevating ATF3 expression through the JNK and p38 pathways [26]. Inhibition of ATF3 has been shown to prevent ferroptosis and alleviate septic lung injury [27]. However, some studies have concluded that ATF3 also exerts anti-inflammatory effects. For instance, ATF3 mitigated pseudomonas aeruginosa-induced ALI in mice; conversely, the deletion of ATF3 resulted in increased concentrations of TNF-α, IL-6, and IL-1β, along with heightened NF-κB activity in lung tissues [28]. The pro-inflammatory effects of ATF3 might outweigh its anti-inflammatory properties in lung tissues following DHCA. This imbalance could exacerbate inflammatory responses and promote cell death, thereby contributing to lung injury after DHCA. Our findings also suggested that ATF3 may serve as an upstream regulatory factor influencing hub gene alterations following DHCA. This observation underscores the potential significance of ATF3 in relation to lung injury subsequent to DHCA.

NR4A1 is an early response gene that belongs to the nuclear hormone receptor superfamily, for which no endogenous ligand has yet been identified. It plays a role in various biological processes, including cell proliferation, apoptosis, inflammation, immunity, and metabolism [17]. It has been suggested that NR4A1 may promote inflammatory responses under certain conditions. For instance, Belgapten inhibited AHR and inflammation in asthma models by targeting NR4A1 to suppress airway inflammation and mast cell activation [29]. Furthermore, NR4A1 appears to facilitate LPS-induced ALI via inhibition of mitochondrial fusion and promotion of necrosis [30]. However, NR4A1 can also mitigate inflammatory responses and maintain immune homeostasis by inhibiting the activation of NF-κB [31]. Additionally, stilbene has been shown to reduce LPS-induced ALI through the activation of NR4A1 [32]. Despite the dual role of NR4A1 in exhibiting both pro-inflammatory and anti-inflammatory effects, we hypothesized that in the context of DHCA, NR4A1 predominantly exerted its pro-inflammatory effects, ultimately contributing to lung injury following DHCA.

CCN1, also known as cysteine-rich protein 61 (CYR61), is a member of the CCN family of stromal cell proteins. These are secreted proteins encoded by early immediate-response genes and play crucial roles in various biological processes, including cell proliferation, differentiation, apoptosis, inflammation, angiogenesis, wound healing, and fibrosis [18]. CCN1 may exert pro-inflammatory effects. For example, alveolar expression of CCN1 correlates with mechanical stretch and the severity of ARDS [33]. Furthermore, CCN1 can be upregulated in ALI models and exacerbates ALI in mice [34]. However, some studies indicate that CCN1 exhibits anti-inflammatory properties. For instance, the matrix protein CCN1—induced by bacterial DNA and CpG ODN—limits lung inflammation and contributes to the maintenance of innate immune homeostasis [35]. Additionally, Astragaloside IV has been shown to ameliorate pulmonary hypertension by enhancing CCN1 expression [36]. We proposed that CCN1 was significantly upregulated in lung tissue following DHCA and primarily contributes to lung injury by exacerbating inflammation and inducing pulmonary hypertension.

ZFP36 is an RNA-binding protein that plays a crucial role in regulating transcriptional processes and inhibiting inflammatory responses. It mitigates mitochondrial damage, oxidative stress, and neuronal apoptosis induced by oxygen-glucose deprivation/reoxygenation through the inhibition of the NOX4-DRP1 pathway [37]. Additionally, ZFP36 protects lung tissue from damage and fibrosis resulting from intestinal ischemia/reperfusion, thereby reducing inflammation-related lung injury [38]. In a cigarette-induced COPD mouse model, ZFP36 was found to decrease emphysema, airway remodeling, and impairment of lung function [38]. However, the increase in ZFP36 levels within lung tissue following DHCA was found to be less pronounced than that of other pro-injury factors. Consequently, its protective effect may be limited. The cumulative impact of various factors ultimately contributes to lung injury after DHCA.

GO analysis revealed that the key genes were associated with biological functions such as cellular responses to external stimuli, intracellular DNA and RNA activation, and cell cycle regulation. KEGG analysis further demonstrated that inflammation pathways related to NF-κB, TNF-α, and IL-17, oxidative stress-related pathways, pathways involved in cell proliferation and apoptosis, as well as B-cell and T-cell related signaling pathways, were upregulated in lung tissues following DHCA. Besides, the PPI results indicated interactions among the key genes. These findings aligned with previous studies. For instance, inflammatory factors such as NF-κB, TNF-α, IL-8, and IL-6 exhibited significant elevation in lung tissues after DHCA compared to pre-reperfusion levels; early activation of NF-κB may play a crucial role in DHCA-induced lung injury [39]. ATF3 and NR4A1 may modulate the inflammatory response by inhibiting NF-κB activity along with the release of TNF-α, IL-6, and IL-1β [28,31]. Additionally, CCN1 enhances macrophage adhesion, activates NF-κB-mediated transcription, and induces the production of pro-inflammatory cytokines by activated M1 macrophages [35]. AP-1 in retinal vascular endothelial cells has been shown to induce the expression of CCN1 protein under hypoxic conditions [40], suggesting an interaction between AP-1 and CCN1. The activation of B and T immune cells may also be influenced by inflammation, oxidative stress, and apoptotic signaling pathways, while interacting with key upregulated genes. For instance, it has been demonstrated that ATF3 regulates the expression of various genes associated with inflammatory responses in both immune and non-immune cells [41]. The EGR1-related signaling pathway is implicated in Th2 cell-associated inflammatory responses observed in allergic dermatoses [42]. CD39 + regulatory T (Treg) cells can modulate autophagy and activate the ERK1/2-FOS pathway to mitigate LPS-induced ALI [43]; thus, an overtransfer of CD39 + Treg cells may represent a therapeutic strategy for preventing and treating ARDS and ALI.

Therefore, the eight hub genes mentioned above might exhibit an imbalance in relation to lung injury and lung protection function through mechanisms involving inflammatory responses, oxidative stress, cellular apoptosis, and immune-related pathways. This imbalance ultimately contributes to lung injury following DHCA. Furthermore, ROC analysis indicated that these hub genes possess high efficacy in predicting genes changes in lung tissues post-DHCA. Consequently, they might serve as valuable biomarkers for assessing potential lung injury after DHCA. As a result, strategies such as inhibiting the target genes, suppressing the inflammatory response, and modulating inflammation-related cellular immunity may help mitigate the inflammatory response and apoptosis of alveolar epithelial cells as well as pulmonary vascular endothelial cells. Additionally, this approach might enhance the balance between pulmonary vasodilation and contraction, thereby reducing lung injury subsequent to DHCA.

Our study established a rat model of DHCA to investigate the lung transcriptomic response following this condition. We identified eight hub genes that were significantly up-regulated, analyzed the major functional pathways associated with these hub genes, and analyzed potential mechanisms underlying DHCA-related lung injury from the perspective of DHCA itself. However, this study has certain limitations: First, as it was conducted using a rat model, further validation is required to determine whether these findings can be directly generalized to humans. Second, the sample size for sequencing in this study was relatively small, which may introduce statistical bias and may result in the exclusion of important genes that exhibit relatively minor differences. Third, we did not validate the relationship between the hub genes and their relevant pathways; thus, the precise roles of these hub genes in lung tissues post-DHCA and their associated molecular mechanisms require further elucidation through additional functional experiments. Furthermore, evaluating the potential clinical applications of these hub genes as biomarkers remains necessary.

## 5. Conclusion

DHCA significantly altered gene expression in rat lung tissues. Inflammation and apoptosis-related genes and signaling pathways may play crucial roles in DHCA-associated lung injury. These findings enhance our understanding of the biological effects of DHCA and offer potential therapeutic targets for protecting lungs from DHCA-related injury.

## Supporting information

S1 Fig Construction of a rat DHCA model. DHCA: deep hypothermic circulatory arrest.
(PNG)

S2 Fig Functional enrichment analysis of eight hub genes. (A) GO enrichment analysis of eight hub genes. (B) KEGG pathway analysis of eight hub genes. GO: Gene Ontology; KEGG: Kyoto Encyclopedia of Genes and Genomes.
(TIF)

S3 Fig ROC curves assessing the predictive capability of each hub gene in predicting lung gene alterations following DHCA. ROC: Receiver Operating Characteristic.
(TIF)

S1 Table The primer sequences for eight rat hub genes. (DOCX)

S2 Table A total of 438 DEGs identified using the DESeq2 method. DEGs: differentially expressed genes.
(DOCX)

S3 Table The specific genes presented in the blue module and the green module identified through WGCNA. WGCNA: Weighted gene co-expression network analysis.
(DOCX)

S4 Table The MCC scores of eight hub genes. MCC: Maximal Clique Centrality.
(DOCX)

S1 File R scripts for the WGCNA analysis. (DOCX)
