# Supplementary figures and images for: Transcriptome profiling and weighted gene co-expression network analysis reveal changes of hub genes and molecular pathways in rat lungs following deep hypothermic circulatory arrest
Source: PLoS One. 2025 Aug 14;20(8):e0328887. doi: 10.1371/journal.pone.0328887 (PMC12352637; doi:10.1371/journal.pone.0328887)

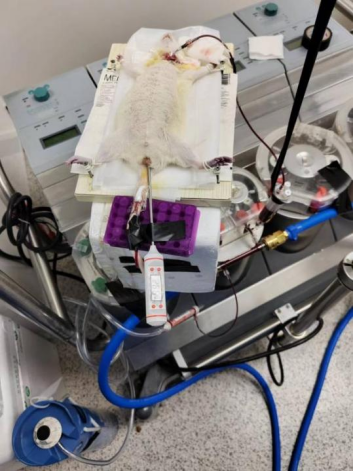

Supplement: S1 Fig — DHCA: deep hypothermic circulatory arrest. (PNG) [file pone.0328887.s001.png]

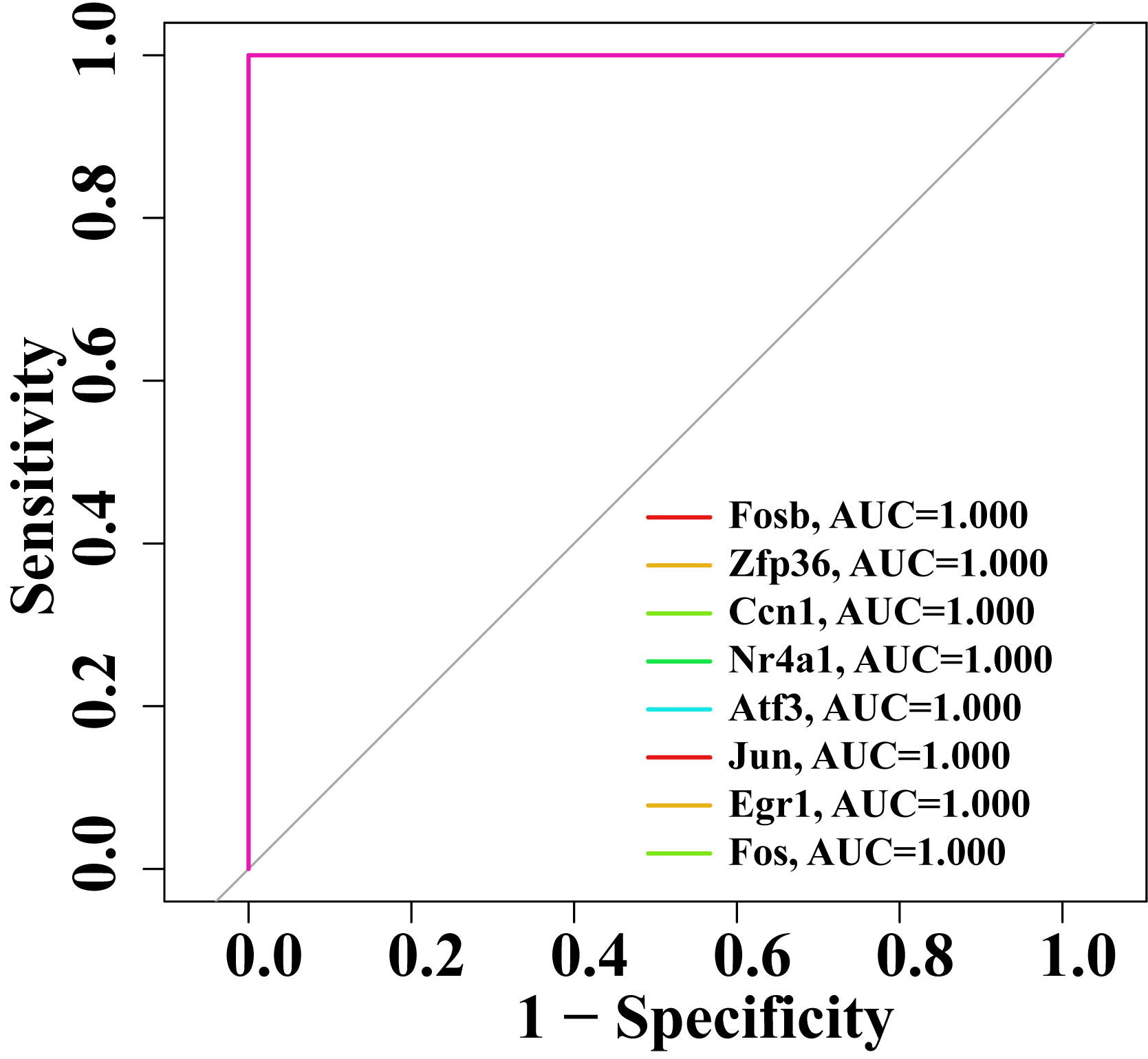

Supplement: S3 Fig — ROC: Receiver Operating Characteristic. (TIF) [file pone.0328887.s003.tif]
