# Supplementary material for: Transcriptome profiling and weighted gene co-expression network analysis reveal changes of hub genes and molecular pathways in rat lungs following deep hypothermic circulatory arrest
Source: PLoS One. 2025 Aug 14;20(8):e0328887. doi: 10.1371/journal.pone.0328887 (PMC12352637; doi:10.1371/journal.pone.0328887)
Supplement: S1 Table — (DOCX) [file pone.0328887.s004.docx]

# S1 Table. **The primer sequences for eight rat hub genes.**

|  | Forward primer sequence | Reverse primer sequence |
| --- | --- | --- |
| FOS | CTGACAGCCACGACAGCATC | TCGGGGTAGGTGAGGTGAGT |
| EGR1 | CCTACGAGCACCTGACCAAG | GCTGCTGTTGCTGAGGTAGA |
| JUN | CGCACGCTCCTAAACAAACTTTG | GTCGTTTCCATCTTTGCAGTCAT |
| ATF3 | AAGAGCTGAGATTCGCCATCC | ACTTGGCAGCAGCAATTTTGT |
| NR4A1 | TCTGCGACCTCTTCCTCTTC | GCTGGTGGTCTTCAGCTTCT |
| CCN1 | GGAAGGTCTACGAGCTGCTG | TGTTCTTGCGGTCATAGCCA |
| ZFP36 | TCTGCCATCTACGAGAGCCTTAT | GGAGTCCGATGAGTTTATGTTCCA |
| FOSB | CAGCCCTGGAGACCTTCAAC | GTCCTTGGAGGTGGCGTAGT |
