# Supplementary material for: Transcriptome profiling and weighted gene co-expression network analysis reveal changes of hub genes and molecular pathways in rat lungs following deep hypothermic circulatory arrest
Source: PLoS One. 2025 Aug 14;20(8):e0328887. doi: 10.1371/journal.pone.0328887 (PMC12352637; doi:10.1371/journal.pone.0328887)
Supplement: S2 Table — DEGs: differentially expressed genes. (DOCX) [file pone.0328887.s005.docx]

**S2 Table. A total of 438 DEGs identified using the DESeq2 method**

|  | Genes | Change | Log2FoldChange | Adjusted P value |
| --- | --- | --- | --- | --- |
| 1 | Fam71a | up-regulated | 9.351 | ＜0.001 |
| 2 | MSTRG.5612 | up-regulated | 8.68 | ＜0.001 |
| 3 | Cxcl2 | up-regulated | 7.581 | ＜0.001 |
| 4 | MSTRG.29244 | up-regulated | 7.528 | 0.001 |
| 5 | MSTRG.15099 | up-regulated | 7.439 | 0.002 |
| 6 | Gm4131 | up-regulated | 7.326 | ＜0.001 |
| 7 | MSTRG.12777 | up-regulated | 7.296 | ＜0.001 |
| 8 | MSTRG.556 | up-regulated | 6.911 | 0.005 |
| 9 | MSTRG.20910 | up-regulated | 6.843 | 0.045 |
| 10 | MSTRG.9514 | up-regulated | 6.652 | ＜0.001 |
| 11 | Tigit | up-regulated | 6.573 | 0.012 |
| 12 | MSTRG.22273 | up-regulated | 6.427 | 0.013 |
| 13 | MSTRG.28659 | up-regulated | 6.417 | 0.03 |
| 14 | Apold1 | up-regulated | 6.308 | ＜0.001 |
| 15 | MSTRG.5611 | up-regulated | 5.911 | 0.005 |
| 16 | MSTRG.27816 | up-regulated | 5.631 | ＜0.001 |
| 17 | MSTRG.29374 | up-regulated | 5.394 | 0.002 |
| 18 | MSTRG.6019 | up-regulated | 5.294 | 0.014 |
| 19 | Pou3f3 | up-regulated | 5.261 | 0.046 |
| 20 | MSTRG.19982 | up-regulated | 5.244 | 0.005 |
| 21 | MSTRG.20484 | up-regulated | 5.233 | ＜0.001 |
| 22 | MSTRG.20290 | up-regulated | 5.066 | 0.038 |
| 23 | MSTRG.10665 | up-regulated | 5.012 | ＜0.001 |
| 24 | Snx31 | up-regulated | 4.975 | ＜0.001 |
| 25 | MSTRG.130 | up-regulated | 4.973 | 0.018 |
| 26 | Cyp2d2 | up-regulated | 4.721 | 0.005 |
| 27 | MSTRG.10442 | up-regulated | 4.656 | 0.006 |
| 28 | MSTRG.12154 | up-regulated | 4.558 | 0.017 |
| 29 | Il1r2 | up-regulated | 4.553 | 0.012 |
| 30 | Pck1 | up-regulated | 4.536 | ＜0.001 |
| 31 | Depp1 | up-regulated | 4.533 | ＜0.001 |
| 32 | Fosb | up-regulated | 4.274 | ＜0.001 |
| 33 | Mt2A | up-regulated | 4.262 | ＜0.001 |
| 34 | Kb23 | up-regulated | 4.26 | 0.032 |
| 35 | Fos | up-regulated | 4.203 | ＜0.001 |
| 36 | MSTRG.10970 | up-regulated | 4.194 | 0.011 |
| 37 | Tnfaip3 | up-regulated | 4.179 | ＜0.001 |
| 38 | Krt17 | up-regulated | 4.113 | 0.048 |
| 39 | MSTRG.8668 | up-regulated | 4.099 | ＜0.001 |
| 40 | Fam163b | up-regulated | 4.086 | 0.005 |
| 41 | MSTRG.1996 | up-regulated | 4.034 | ＜0.001 |
| 42 | MSTRG.9963 | up-regulated | 3.999 | 0.005 |
| 43 | Rgs1 | up-regulated | 3.962 | ＜0.001 |
| 44 | Atf3 | up-regulated | 3.951 | 0.007 |
| 45 | MSTRG.25412 | up-regulated | 3.828 | 0.008 |
| 46 | Krt83 | up-regulated | 3.822 | 0.004 |
| 47 | Tenm1 | up-regulated | 3.787 | 0.049 |
| 48 | MSTRG.2181 | up-regulated | 3.764 | ＜0.001 |
| 49 | MSTRG.15268 | up-regulated | 3.648 | 0.043 |
| 50 | Cxcl1 | up-regulated | 3.586 | 0.001 |
| 51 | MSTRG.19002 | up-regulated | 3.559 | ＜0.001 |
| 52 | Il1a | up-regulated | 3.474 | 0.004 |
| 53 | Olr1387 | up-regulated | 3.467 | 0.007 |
| 54 | MSTRG.17193 | up-regulated | 3.462 | 0.036 |
| 55 | Hist1h2af | up-regulated | 3.459 | 0.031 |
| 56 | Egr1 | up-regulated | 3.396 | ＜0.001 |
| 57 | AC128848.1 | up-regulated | 3.377 | 0.009 |
| 58 | Olr1565 | up-regulated | 3.376 | 0.026 |
| 59 | Errfi1 | up-regulated | 3.307 | ＜0.001 |
| 60 | Trim66 | up-regulated | 3.304 | ＜0.001 |
| 61 | Il10 | up-regulated | 3.29 | 0.009 |
| 62 | Sik1 | up-regulated | 3.27 | ＜0.001 |
| 63 | MSTRG.6694 | up-regulated | 3.246 | 0.001 |
| 64 | MSTRG.26618 | up-regulated | 3.18 | ＜0.001 |
| 65 | Nr4a3 | up-regulated | 3.178 | ＜0.001 |
| 66 | Osm | up-regulated | 3.139 | ＜0.001 |
| 67 | Nfil3 | up-regulated | 3.118 | ＜0.001 |
| 68 | MSTRG.6292 | up-regulated | 3.099 | 0.018 |
| 69 | Syt1 | up-regulated | 3.085 | 0.035 |
| 70 | H1f4 | up-regulated | 3.082 | 0.011 |
| 71 | MSTRG.21562 | up-regulated | 3.079 | 0.003 |
| 72 | Nr4a1 | up-regulated | 3.075 | ＜0.001 |
| 73 | Sphk1 | up-regulated | 3.068 | ＜0.001 |
| 74 | MSTRG.25505 | up-regulated | 3.016 | ＜0.001 |
| 75 | Erp27 | up-regulated | 3.015 | 0.014 |
| 76 | Ldhc | up-regulated | 2.998 | 0.005 |
| 77 | AABR07021465.1 | up-regulated | 2.987 | 0.004 |
| 78 | Il1b | up-regulated | 2.944 | 0.047 |
| 79 | MSTRG.12243 | up-regulated | 2.92 | 0.043 |
| 80 | MSTRG.29246 | up-regulated | 2.882 | 0.019 |
| 81 | MSTRG.21610 | up-regulated | 2.878 | ＜0.001 |
| 82 | Slc5a3 | up-regulated | 2.861 | 0.001 |
| 83 | MSTRG.24698 | up-regulated | 2.849 | ＜0.001 |
| 84 | MSTRG.27405 | up-regulated | 2.816 | ＜0.001 |
| 85 | MSTRG.29373 | up-regulated | 2.79 | ＜0.001 |
| 86 | Lrtm2 | up-regulated | 2.776 | 0.015 |
| 87 | MSTRG.21710 | up-regulated | 2.764 | 0.005 |
| 88 | MSTRG.29127 | up-regulated | 2.733 | 0.007 |
| 89 | MSTRG.33865 | up-regulated | 2.706 | 0.013 |
| 90 | Map3k8 | up-regulated | 2.699 | ＜0.001 |
| 91 | Mbl1 | up-regulated | 2.695 | 0.003 |
| 92 | MSTRG.26270 | up-regulated | 2.691 | 0.007 |
| 93 | Cxcl10 | up-regulated | 2.685 | ＜0.001 |
| 94 | Edn1 | up-regulated | 2.676 | 0.008 |
| 95 | Ptgs2 | up-regulated | 2.626 | ＜0.001 |
| 96 | MSTRG.10670 | up-regulated | 2.579 | 0.009 |
| 97 | MSTRG.33538 | up-regulated | 2.562 | 0.009 |
| 98 | MSTRG.8809 | up-regulated | 2.522 | 0.009 |
| 99 | Rel | up-regulated | 2.512 | 0.001 |
| 100 | Mt1 | up-regulated | 2.492 | 0.019 |
| 101 | Serpine1 | up-regulated | 2.479 | 0.027 |
| 102 | MSTRG.18848 | up-regulated | 2.472 | 0.02 |
| 103 | Zfp36 | up-regulated | 2.469 | ＜0.001 |
| 104 | MSTRG.17435 | up-regulated | 2.455 | 0.001 |
| 105 | Ppp1r15a | up-regulated | 2.45 | 0.035 |
| 106 | Ccn1 | up-regulated | 2.445 | 0.022 |
| 107 | Penk | up-regulated | 2.441 | ＜0.001 |
| 108 | Ccl3 | up-regulated | 2.416 | ＜0.001 |
| 109 | Hsf2bp | up-regulated | 2.379 | 0.011 |
| 110 | MSTRG.15031 | up-regulated | 2.322 | 0.001 |
| 111 | Sdcbp2 | up-regulated | 2.279 | ＜0.001 |
| 112 | RGD1562811 | up-regulated | 2.278 | 0.002 |
| 113 | Pde4d | up-regulated | 2.253 | 0.004 |
| 114 | Birc3 | up-regulated | 2.233 | 0.001 |
| 115 | Creb5 | up-regulated | 2.226 | 0.022 |
| 116 | MSTRG.16080 | up-regulated | 2.214 | 0.012 |
| 117 | Tmem252 | up-regulated | 2.197 | ＜0.001 |
| 118 | F3 | up-regulated | 2.196 | ＜0.001 |
| 119 | MSTRG.29126 | up-regulated | 2.19 | ＜0.001 |
| 120 | MSTRG.22611 | up-regulated | 2.189 | 0.008 |
| 121 | Cdkn1a | up-regulated | 2.185 | ＜0.001 |
| 122 | MSTRG.11803 | up-regulated | 2.182 | ＜0.001 |
| 123 | MSTRG.4327 | up-regulated | 2.176 | 0.012 |
| 124 | MSTRG.25739 | up-regulated | 2.174 | ＜0.001 |
| 125 | Mefv | up-regulated | 2.17 | 0.001 |
| 126 | Rasd1 | up-regulated | 2.168 | ＜0.001 |
| 127 | Sele | up-regulated | 2.165 | 0.012 |
| 128 | Dusp10 | up-regulated | 2.146 | ＜0.001 |
| 129 | Col19a1 | up-regulated | 2.144 | 0.016 |
| 130 | Mfsd2a | up-regulated | 2.14 | ＜0.001 |
| 131 | Hist2h3c2 | up-regulated | 2.139 | ＜0.001 |
| 132 | MSTRG.34461 | up-regulated | 2.134 | ＜0.001 |
| 133 | Rab44 | up-regulated | 2.128 | 0.045 |
| 134 | Lif | up-regulated | 2.121 | 0.03 |
| 135 | Dbh | up-regulated | 2.105 | 0.026 |
| 136 | Nfkbia | up-regulated | 2.101 | ＜0.001 |
| 137 | Nfkbiz | up-regulated | 2.097 | ＜0.001 |
| 138 | MSTRG.10519 | up-regulated | 2.092 | 0.005 |
| 139 | MSTRG.23698 | up-regulated | 2.092 | 0.022 |
| 140 | Csrnp1 | up-regulated | 2.074 | ＜0.001 |
| 141 | MSTRG.29370 | up-regulated | 2.07 | 0.001 |
| 142 | Gadd45g | up-regulated | 2.057 | ＜0.001 |
| 143 | MSTRG.31971 | up-regulated | 2.043 | 0.012 |
| 144 | Col10a1 | up-regulated | 2.024 | 0.038 |
| 145 | Dusp13 | up-regulated | 2.022 | 0.041 |
| 146 | AABR07065031.1 | up-regulated | 2.017 | 0.006 |
| 147 | Socs3 | up-regulated | 2.011 | 0.001 |
| 148 | Pfkfb3 | up-regulated | 2.004 | ＜0.001 |
| 149 | Lrrc32 | up-regulated | 2.003 | ＜0.001 |
| 150 | Sema7a | up-regulated | 2 | ＜0.001 |
| 151 | MSTRG.30141 | up-regulated | 1.977 | 0.012 |
| 152 | Pmaip1 | up-regulated | 1.958 | ＜0.001 |
| 153 | MSTRG.13607 | up-regulated | 1.951 | 0.04 |
| 154 | Trim54 | up-regulated | 1.946 | 0.001 |
| 155 | MSTRG.226 | up-regulated | 1.946 | ＜0.001 |
| 156 | Icam4 | up-regulated | 1.93 | 0.021 |
| 157 | Pde4b | up-regulated | 1.928 | 0.004 |
| 158 | Ank1 | up-regulated | 1.923 | 0.005 |
| 159 | MSTRG.29917 | up-regulated | 1.918 | 0.004 |
| 160 | Ribc2 | up-regulated | 1.885 | 0.032 |
| 161 | Per1 | up-regulated | 1.881 | ＜0.001 |
| 162 | MSTRG.32904 | up-regulated | 1.871 | 0.049 |
| 163 | Ccdc33 | up-regulated | 1.865 | 0.019 |
| 164 | MSTRG.26256 | up-regulated | 1.836 | 0.009 |
| 165 | MSTRG.3219 | up-regulated | 1.825 | 0.019 |
| 166 | Tnf | up-regulated | 1.824 | 0.05 |
| 167 | MSTRG.15920 | up-regulated | 1.817 | 0.025 |
| 168 | MSTRG.3555 | up-regulated | 1.812 | 0.001 |
| 169 | Map3k6 | up-regulated | 1.811 | ＜0.001 |
| 170 | Sh2d2a | up-regulated | 1.81 | ＜0.001 |
| 171 | Insm1 | up-regulated | 1.809 | 0.014 |
| 172 | MSTRG.28056 | up-regulated | 1.797 | 0.04 |
| 173 | Adamts1 | up-regulated | 1.777 | ＜0.001 |
| 174 | Serpind1 | up-regulated | 1.773 | 0.022 |
| 175 | C2cd4b | up-regulated | 1.743 | 0.042 |
| 176 | Slc30a3 | up-regulated | 1.719 | 0.003 |
| 177 | Hcar2 | up-regulated | 1.717 | 0.047 |
| 178 | Ankrd37 | up-regulated | 1.712 | 0.031 |
| 179 | Arl4a | up-regulated | 1.71 | 0.005 |
| 180 | Acr | up-regulated | 1.7 | ＜0.001 |
| 181 | Mideas | up-regulated | 1.698 | 0.022 |
| 182 | Traf6 | up-regulated | 1.697 | 0.04 |
| 183 | Ccl4 | up-regulated | 1.687 | 0.012 |
| 184 | MSTRG.12422 | up-regulated | 1.683 | 0.014 |
| 185 | Adamts9 | up-regulated | 1.682 | 0.002 |
| 186 | Mab21l4 | up-regulated | 1.674 | ＜0.001 |
| 187 | Lilrb2 | up-regulated | 1.673 | 0.008 |
| 188 | Nuak2 | up-regulated | 1.669 | 0.005 |
| 189 | MSTRG.6690 | up-regulated | 1.65 | ＜0.001 |
| 190 | Btg2 | up-regulated | 1.648 | ＜0.001 |
| 191 | Tbc1d8 | up-regulated | 1.647 | ＜0.001 |
| 192 | Gpr132 | up-regulated | 1.639 | 0.039 |
| 193 | Trim16 | up-regulated | 1.629 | 0.003 |
| 194 | Jun | up-regulated | 1.627 | 0.002 |
| 195 | Trib3 | up-regulated | 1.622 | ＜0.001 |
| 196 | MSTRG.19927 | up-regulated | 1.619 | 0.019 |
| 197 | Aff1 | up-regulated | 1.596 | 0.03 |
| 198 | Bcl3 | up-regulated | 1.591 | 0.036 |
| 199 | Adora2b | up-regulated | 1.586 | 0.022 |
| 200 | MSTRG.5584 | up-regulated | 1.582 | ＜0.001 |
| 201 | RGD1560146 | up-regulated | 1.578 | 0.021 |
| 202 | Arrdc3 | up-regulated | 1.577 | ＜0.001 |
| 203 | Dynlt4 | up-regulated | 1.576 | 0.008 |
| 204 | Aspa | up-regulated | 1.574 | 0.004 |
| 205 | Nfkb2 | up-regulated | 1.57 | 0.009 |
| 206 | Crem | up-regulated | 1.556 | ＜0.001 |
| 207 | MSTRG.33277 | up-regulated | 1.555 | 0.042 |
| 208 | Noct | up-regulated | 1.548 | 0.044 |
| 209 | AABR07008066.2 | up-regulated | 1.539 | 0.013 |
| 210 | Dusp16 | up-regulated | 1.525 | 0.033 |
| 211 | Slc26a9 | up-regulated | 1.525 | 0.046 |
| 212 | Plat | up-regulated | 1.521 | 0.005 |
| 213 | MSTRG.1249 | up-regulated | 1.52 | ＜0.001 |
| 214 | Gem | up-regulated | 1.512 | 0.002 |
| 215 | MSTRG.21615 | up-regulated | 1.509 | 0.001 |
| 216 | MSTRG.3977 | up-regulated | 1.5 | 0.012 |
| 217 | MSTRG.24061 | up-regulated | 1.479 | 0.03 |
| 218 | Dusp2 | up-regulated | 1.476 | 0.009 |
| 219 | MSTRG.13342 | up-regulated | 1.475 | 0.028 |
| 220 | Cish | up-regulated | 1.469 | 0.003 |
| 221 | Pim1 | up-regulated | 1.466 | 0.005 |
| 222 | Hap1 | up-regulated | 1.465 | 0.004 |
| 223 | Cd69 | up-regulated | 1.452 | 0.031 |
| 224 | Clec18a | up-regulated | 1.439 | 0.028 |
| 225 | MSTRG.29179 | up-regulated | 1.428 | 0.043 |
| 226 | Prr7 | up-regulated | 1.425 | 0.045 |
| 227 | Coq10b | up-regulated | 1.424 | 0.02 |
| 228 | MSTRG.25067 | up-regulated | 1.422 | 0.006 |
| 229 | Fgfbp1 | up-regulated | 1.406 | 0.004 |
| 230 | Rgs16 | up-regulated | 1.402 | 0.004 |
| 231 | Il4r | up-regulated | 1.396 | ＜0.001 |
| 232 | Dnai1 | up-regulated | 1.396 | 0.005 |
| 233 | Sidt1 | up-regulated | 1.393 | 0.027 |
| 234 | Ccdc60 | up-regulated | 1.391 | 0.034 |
| 235 | MSTRG.9735 | up-regulated | 1.39 | 0.013 |
| 236 | Lax1 | up-regulated | 1.39 | 0.005 |
| 237 | NEWGENE_1309147 | up-regulated | 1.39 | 0.039 |
| 238 | MSTRG.16861 | up-regulated | 1.387 | 0.001 |
| 239 | Cldn4 | up-regulated | 1.38 | 0.002 |
| 240 | MSTRG.16907 | up-regulated | 1.376 | ＜0.001 |
| 241 | Tbxt | up-regulated | 1.37 | 0.001 |
| 242 | Cblb | up-regulated | 1.366 | 0.03 |
| 243 | Cdc42ep2 | up-regulated | 1.354 | ＜0.001 |
| 244 | Cfap91 | up-regulated | 1.338 | 0.001 |
| 245 | MSTRG.7417 | up-regulated | 1.332 | 0.009 |
| 246 | Efhb | up-regulated | 1.327 | ＜0.001 |
| 247 | MSTRG.26711 | up-regulated | 1.324 | 0.004 |
| 248 | Nfkbib | up-regulated | 1.32 | 0.05 |
| 249 | Ackr3 | up-regulated | 1.32 | ＜0.001 |
| 250 | Tfcp2l1 | up-regulated | 1.318 | 0.03 |
| 251 | Fndc1 | up-regulated | 1.312 | 0.011 |
| 252 | Fabp4 | up-regulated | 1.302 | 0.011 |
| 253 | Ier3 | up-regulated | 1.298 | 0.026 |
| 254 | Cebpd | up-regulated | 1.286 | 0.003 |
| 255 | Capsl | up-regulated | 1.283 | 0.001 |
| 256 | Vsig2 | up-regulated | 1.282 | 0.019 |
| 257 | MSTRG.29861 | up-regulated | 1.277 | 0.006 |
| 258 | Sema4c | up-regulated | 1.273 | 0.002 |
| 259 | Cfap73 | up-regulated | 1.271 | 0.041 |
| 260 | MSTRG.16454 | up-regulated | 1.264 | 0.005 |
| 261 | MSTRG.26782 | up-regulated | 1.263 | 0.002 |
| 262 | MSTRG.23656 | up-regulated | 1.261 | 0.006 |
| 263 | Irak2 | up-regulated | 1.245 | 0.003 |
| 264 | Il7r | up-regulated | 1.242 | 0.008 |
| 265 | Cd83 | up-regulated | 1.24 | 0.001 |
| 266 | Adam8 | up-regulated | 1.23 | 0.003 |
| 267 | Ier2 | up-regulated | 1.228 | 0.008 |
| 268 | Lrriq4 | up-regulated | 1.222 | 0.049 |
| 269 | Il6r | up-regulated | 1.22 | ＜0.001 |
| 270 | Foxp3 | up-regulated | 1.207 | 0.04 |
| 271 | MSTRG.5403 | up-regulated | 1.203 | 0.014 |
| 272 | Cebpb | up-regulated | 1.198 | 0.012 |
| 273 | Tcte1 | up-regulated | 1.196 | 0.021 |
| 274 | Foxo3 | up-regulated | 1.19 | 0.047 |
| 275 | Dyrk3 | up-regulated | 1.187 | 0.015 |
| 276 | Chrne | up-regulated | 1.186 | 0.002 |
| 277 | Tmem139 | up-regulated | 1.183 | ＜0.001 |
| 278 | Odad3 | up-regulated | 1.181 | 0.049 |
| 279 | Cacna1d | up-regulated | 1.176 | 0.04 |
| 280 | Ifit3 | up-regulated | 1.173 | 0.041 |
| 281 | MSTRG.15341 | up-regulated | 1.172 | 0.01 |
| 282 | MSTRG.23747 | up-regulated | 1.169 | 0.001 |
| 283 | Cfap74 | up-regulated | 1.169 | 0.018 |
| 284 | Relb | up-regulated | 1.168 | 0.019 |
| 285 | Dnajb13 | up-regulated | 1.164 | 0.011 |
| 286 | Txnip | up-regulated | 1.162 | ＜0.001 |
| 287 | Nek5 | up-regulated | 1.148 | ＜0.001 |
| 288 | Proser2 | up-regulated | 1.14 | 0.023 |
| 289 | MSTRG.21393 | up-regulated | 1.137 | 0.009 |
| 290 | Fam222a | up-regulated | 1.136 | 0.044 |
| 291 | Ggt1 | up-regulated | 1.131 | 0.023 |
| 292 | Bcl2l11 | up-regulated | 1.131 | 0.004 |
| 293 | Itprip | up-regulated | 1.125 | 0.014 |
| 294 | Slc7a5 | up-regulated | 1.124 | 0.006 |
| 295 | Ccdc189 | up-regulated | 1.124 | 0.018 |
| 296 | Cysrt1 | up-regulated | 1.122 | 0.04 |
| 297 | Drc7 | up-regulated | 1.122 | 0.049 |
| 298 | Npas2 | up-regulated | 1.119 | 0.023 |
| 299 | Tekt1 | up-regulated | 1.112 | 0.018 |
| 300 | Nr4a2 | up-regulated | 1.107 | 0.011 |
| 301 | Vmp1 | up-regulated | 1.105 | 0.01 |
| 302 | Ubap1l | up-regulated | 1.104 | 0.032 |
| 303 | Mapk15 | up-regulated | 1.102 | 0.029 |
| 304 | Itk | up-regulated | 1.1 | 0.035 |
| 305 | H3f3a | up-regulated | 1.099 | 0.001 |
| 306 | H1f2 | up-regulated | 1.095 | ＜0.001 |
| 307 | MSTRG.26613 | up-regulated | 1.092 | 0.041 |
| 308 | Itga2b | up-regulated | 1.091 | 0.035 |
| 309 | Gpr171 | up-regulated | 1.091 | 0.022 |
| 310 | Sema6b | up-regulated | 1.087 | 0.012 |
| 311 | Ppp1r10 | up-regulated | 1.086 | ＜0.001 |
| 312 | Ccdc180 | up-regulated | 1.086 | 0.021 |
| 313 | Cdhr4 | up-regulated | 1.078 | 0.01 |
| 314 | Fem1b | up-regulated | 1.076 | 0.012 |
| 315 | Bcar3 | up-regulated | 1.072 | 0.022 |
| 316 | Egr2 | up-regulated | 1.072 | 0.026 |
| 317 | Rgcc | up-regulated | 1.071 | 0.038 |
| 318 | Fhdc1 | up-regulated | 1.07 | 0.009 |
| 319 | Slc23a2 | up-regulated | 1.069 | 0.005 |
| 320 | Pim3 | up-regulated | 1.064 | ＜0.001 |
| 321 | Alas1 | up-regulated | 1.055 | 0.015 |
| 322 | Rasgef1b | up-regulated | 1.055 | 0.003 |
| 323 | MSTRG.6586 | up-regulated | 1.054 | 0.05 |
| 324 | Drc3 | up-regulated | 1.05 | 0.041 |
| 325 | Mecp2 | up-regulated | 1.048 | 0.049 |
| 326 | Vcam1 | up-regulated | 1.043 | 0.015 |
| 327 | AABR07071287.1 | up-regulated | 1.034 | 0.043 |
| 328 | MSTRG.31010 | up-regulated | 1.032 | 0.007 |
| 329 | Ccdc162 | up-regulated | 1.029 | 0.005 |
| 330 | AABR07005985.1 | up-regulated | 1.018 | 0.01 |
| 331 | Cfap65 | up-regulated | 1.017 | 0.014 |
| 332 | Cdhr3 | up-regulated | 1.006 | 0.049 |
| 333 | Heyl | up-regulated | 1.006 | 0.038 |
| 334 | MSTRG.439 | up-regulated | 1.004 | 0.03 |
| 335 | MSTRG.26027 | up-regulated | 1.004 | ＜0.001 |
| 336 | Dnaaf8 | up-regulated | 1.002 | 0.026 |
| 337 | Cfap43 | up-regulated | 1.002 | 0.02 |
| 338 | Rbm14 | down-regulated | -1 | 0.006 |
| 339 | Cx3cr1 | down-regulated | -1.018 | 0.014 |
| 340 | Zfp60 | down-regulated | -1.031 | 0.014 |
| 341 | Zfp799 | down-regulated | -1.034 | 0.001 |
| 342 | P2ry6 | down-regulated | -1.04 | 0.044 |
| 343 | Rbm12b | down-regulated | -1.044 | 0.004 |
| 344 | Gvin1 | down-regulated | -1.044 | 0.011 |
| 345 | MSTRG.2820 | down-regulated | -1.064 | 0.017 |
| 346 | Spn | down-regulated | -1.066 | 0.001 |
| 347 | Ahr | down-regulated | -1.074 | ＜0.001 |
| 348 | Lzts1 | down-regulated | -1.075 | 0.044 |
| 349 | Gemin7 | down-regulated | -1.076 | 0.045 |
| 350 | Derl3 | down-regulated | -1.079 | 0.01 |
| 351 | Btbd3 | down-regulated | -1.093 | 0.026 |
| 352 | Jrkl | down-regulated | -1.095 | 0.006 |
| 353 | Rrp8 | down-regulated | -1.097 | 0.013 |
| 354 | Xkr8 | down-regulated | -1.098 | 0.035 |
| 355 | Rcc1 | down-regulated | -1.098 | 0.001 |
| 356 | Ccn2 | down-regulated | -1.107 | 0.028 |
| 357 | Adrb2 | down-regulated | -1.112 | ＜0.001 |
| 358 | Tmlhe | down-regulated | -1.113 | 0.001 |
| 359 | AABR07055919.1 | down-regulated | -1.136 | 0.005 |
| 360 | Id3 | down-regulated | -1.143 | 0.022 |
| 361 | Cbx2 | down-regulated | -1.144 | 0.006 |
| 362 | Ezh2 | down-regulated | -1.151 | ＜0.001 |
| 363 | Zscan12 | down-regulated | -1.152 | 0.018 |
| 364 | Ccdc69 | down-regulated | -1.154 | 0.038 |
| 365 | Ephb3 | down-regulated | -1.163 | 0.005 |
| 366 | Ttn | down-regulated | -1.172 | 0.043 |
| 367 | Tnfrsf13c | down-regulated | -1.179 | 0.049 |
| 368 | Lpar6 | down-regulated | -1.182 | ＜0.001 |
| 369 | Gimap4 | down-regulated | -1.187 | ＜0.001 |
| 370 | Bambi | down-regulated | -1.196 | 0.01 |
| 371 | Lyl1 | down-regulated | -1.203 | 0.022 |
| 372 | MSTRG.23749 | down-regulated | -1.204 | ＜0.001 |
| 373 | Xrcc2 | down-regulated | -1.211 | 0.032 |
| 374 | Hpgd | down-regulated | -1.214 | 0.011 |
| 375 | Oip5 | down-regulated | -1.223 | 0.027 |
| 376 | MSTRG.21755 | down-regulated | -1.237 | 0.022 |
| 377 | MSTRG.23190 | down-regulated | -1.248 | 0.009 |
| 378 | Cd180 | down-regulated | -1.254 | 0.006 |
| 379 | MSTRG.29699 | down-regulated | -1.258 | 0.015 |
| 380 | RGD1565166 | down-regulated | -1.26 | 0.028 |
| 381 | C17h6orf52 | down-regulated | -1.263 | 0.022 |
| 382 | Slitrk6 | down-regulated | -1.269 | 0.019 |
| 383 | Fam83d | down-regulated | -1.282 | ＜0.001 |
| 384 | Gata3 | down-regulated | -1.301 | 0.006 |
| 385 | Sh3bp5l | down-regulated | -1.303 | ＜0.001 |
| 386 | MSTRG.12415 | down-regulated | -1.305 | 0.049 |
| 387 | Hhex | down-regulated | -1.324 | 0.005 |
| 388 | Hyls1 | down-regulated | -1.325 | ＜0.001 |
| 389 | C3ar1 | down-regulated | -1.331 | 0.001 |
| 390 | Cyp26b1 | down-regulated | -1.351 | 0.001 |
| 391 | RGD1564463 | down-regulated | -1.368 | 0.003 |
| 392 | Eomes | down-regulated | -1.376 | 0.043 |
| 393 | Fzd8 | down-regulated | -1.381 | 0.032 |
| 394 | Setmar | down-regulated | -1.381 | 0.039 |
| 395 | MSTRG.27155 | down-regulated | -1.39 | 0.001 |
| 396 | Gimap9 | down-regulated | -1.391 | ＜0.001 |
| 397 | MSTRG.23821 | down-regulated | -1.394 | 0.023 |
| 398 | MSTRG.20370 | down-regulated | -1.444 | 0.031 |
| 399 | Mxd3 | down-regulated | -1.447 | 0.039 |
| 400 | Zfp111 | down-regulated | -1.463 | 0.042 |
| 401 | Ier5l | down-regulated | -1.471 | 0.018 |
| 402 | Zcchc3 | down-regulated | -1.481 | 0.001 |
| 403 | Pcdh1 | down-regulated | -1.483 | ＜0.001 |
| 404 | S1pr5 | down-regulated | -1.486 | 0.002 |
| 405 | MSTRG.23578 | down-regulated | -1.509 | 0.049 |
| 406 | MSTRG.23300 | down-regulated | -1.52 | ＜0.001 |
| 407 | Amotl2 | down-regulated | -1.521 | ＜0.001 |
| 408 | Sox18 | down-regulated | -1.541 | 0.012 |
| 409 | Zfp623 | down-regulated | -1.551 | 0.002 |
| 410 | MSTRG.10620 | down-regulated | -1.566 | 0.008 |
| 411 | Zfp566 | down-regulated | -1.566 | 0.019 |
| 412 | MSTRG.27157 | down-regulated | -1.57 | 0.002 |
| 413 | Gpr68 | down-regulated | -1.574 | 0.001 |
| 414 | Cyp1a1 | down-regulated | -1.587 | ＜0.001 |
| 415 | Thap6 | down-regulated | -1.596 | ＜0.001 |
| 416 | MSTRG.33350 | down-regulated | -1.621 | 0.017 |
| 417 | MSTRG.23239 | down-regulated | -1.645 | 0.022 |
| 418 | Mylk2 | down-regulated | -1.713 | 0.01 |
| 419 | MSTRG.21634 | down-regulated | -1.757 | 0.037 |
| 420 | Clec4g | down-regulated | -1.776 | 0.001 |
| 421 | MSTRG.15753 | down-regulated | -1.794 | ＜0.001 |
| 422 | Mex3b | down-regulated | -1.827 | ＜0.001 |
| 423 | MSTRG.11008 | down-regulated | -1.912 | 0.021 |
| 424 | Inka1 | down-regulated | -1.94 | 0.001 |
| 425 | Sp5 | down-regulated | -1.995 | 0.015 |
| 426 | MSTRG.102 | down-regulated | -2.067 | 0.034 |
| 427 | MSTRG.25644 | down-regulated | -2.092 | 0.007 |
| 428 | MSTRG.6689 | down-regulated | -2.155 | ＜0.001 |
| 429 | Tnfrsf17 | down-regulated | -2.32 | 0.016 |
| 430 | Mycn | down-regulated | -2.378 | ＜0.001 |
| 431 | AABR07001512.1 | down-regulated | -3.035 | 0.011 |
| 432 | Fcrl2 | down-regulated | -3.175 | 0.006 |
| 433 | MSTRG.24281 | down-regulated | -3.365 | 0.019 |
| 434 | MSTRG.12184 | down-regulated | -3.48 | 0.008 |
| 435 | Gp2 | down-regulated | -4.994 | 0.043 |
| 436 | MSTRG.27919 | down-regulated | -6.865 | 0.001 |
| 437 | RatNP-3b | down-regulated | -7.499 | ＜0.001 |
| 438 | AABR07065823.1 | down-regulated | -9.699 | ＜0.001 |
