# Supplementary material for: Transcriptome profiling and weighted gene co-expression network analysis reveal changes of hub genes and molecular pathways in rat lungs following deep hypothermic circulatory arrest
Source: PLoS One. 2025 Aug 14;20(8):e0328887. doi: 10.1371/journal.pone.0328887 (PMC12352637; doi:10.1371/journal.pone.0328887)
Supplement: S3 Table — WGCNA: Weighted gene co-expression network analysis. (DOCX) [file pone.0328887.s006.docx]

**S3 Table. The specific genes presented in the blue module and the green module identified through WGCNA.**

| hubGenes MMblue (n=197) | hubGenes MMturquoise  (n=212) |
| --- | --- |
| Cdhr3 | Btg2 |
| Cdhr4 | Tbc1d8 |
| MSTRG.26711 | Id3 |
| Lrriq4 | MSTRG.6292 |
| MSTRG.21634 | Dusp16 |
| Lyl1 | Dusp10 |
| MSTRG.439 | Dusp13 |
| Slc23a2 | Noct |
| Hsf2bp | Tmem252 |
| Ccdc33 | Osm |
| Fem1b | MSTRG.8668 |
| AABR07071287.1 | MSTRG.4327 |
| MSTRG.6586 | MSTRG.10442 |
| MSTRG.26613 | MSTRG.8809 |
| MSTRG.26618 | MSTRG.17435 |
| Sh3bp5l | MSTRG.26270 |
| MSTRG.15031 | Xkr8 |
| Rbm12b | Arl4a |
| MSTRG.24698 | Errfi1 |
| Alas1 | Gpr132 |
| Tnfrsf13c | MSTRG.25067 |
| Il6r | MSTRG.20484 |
| MSTRG.1249 | Tnf |
| Rgs1 | Nfkbib |
| Rbm14 | Nfkbiz |
| Nfkbia | Birc3 |
| Mxd3 | MSTRG.29699 |
| Thap6 | Heyl |
| Ackr3 | Cdkn1a |
| Penk | MSTRG.10519 |
| S1pr5 | Dusp2 |
| Cfap91 | Fhdc1 |
| Txnip | RGD1564463 |
| Mab21l4 | MSTRG.2181 |
| Slc5a3 | Relb |
| MSTRG.30141 | MSTRG.9735 |
| MSTRG.11008 | Rgcc |
| Slitrk6 | MSTRG.20290 |
| Col10a1 | Nuak2 |
| Gemin7 | MSTRG.6694 |
| Tmem139 | Slc7a5 |
| C17h6orf52 | Tenm1 |
| Cbx2 | Ppp1r15a |
| Ppp1r10 | MSTRG.19982 |
| MSTRG.6690 | Rab44 |
| MSTRG.31971 | MSTRG.10620 |
| MSTRG.23821 | Nr4a2 |
| Mapk15 | Nr4a3 |
| Adam8 | MSTRG.25412 |
| Cysrt1 | Edn1 |
| MSTRG.10670 | MSTRG.7417 |
| Sidt1 | Ephb3 |
| Ccdc189 | Trim16 |
| Serpine1 | Dyrk3 |
| Lzts1 | Kb23 |
| Aff1 | MSTRG.9963 |
| Mycn | Map3k8 |
| AABR07055919.1 | Map3k6 |
| Foxp3 | RGD1562811 |
| Dynlt4 | Tfcp2l1 |
| Rgs16 | Cdc42ep2 |
| MSTRG.5584 | Lrrc32 |
| Cfap65 | Il10 |
| Foxo3 | MSTRG.29374 |
| MSTRG.33350 | MSTRG.29373 |
| MSTRG.15753 | MSTRG.29370 |
| Odad3 | MSTRG.15268 |
| MSTRG.21755 | MSTRG.33538 |
| MSTRG.24061 | Gem |
| Fos | Arrdc3 |
| Drc7 | Hcar2 |
| Drc3 | Bcl3 |
| Nek5 | Krt83 |
| MSTRG.23656 | MSTRG.17193 |
| Hyls1 | Cxcl1 |
| MSTRG.26027 | MSTRG.29917 |
| Clec4g | Lrtm2 |
| Ccdc180 | MSTRG.26782 |
| AABR07001512.1 | Coq10b |
| MSTRG.26256 | MSTRG.556 |
| Nr4a1 | Sema6b |
| MSTRG.20370 | MSTRG.15099 |
| C3ar1 | Pde4b |
| Pde4d | Nfkb2 |
| MSTRG.33865 | Cish |
| Adamts1 | MSTRG.12243 |
| Adamts9 | Sp5 |
| Trim66 | Ahr |
| Rasgef1b | Prr7 |
| Spn | Mideas |
| MSTRG.19002 | Bcar3 |
| Mfsd2a | Cxcl2 |
| MSTRG.23190 | Cxcl10 |
| MSTRG.12777 | Egr2 |
| MSTRG.16907 | Sdcbp2 |
| Zfp623 | Inka1 |
| Rcc1 | MSTRG.22273 |
| MSTRG.29126 | MSTRG.3977 |
| MSTRG.29127 | Il1r2 |
| Egr1 | Gata3 |
| MSTRG.25739 | MSTRG.18848 |
| MSTRG.19927 | Zfp566 |
| Fam222a | Gm4131 |
| MSTRG.11803 | Vsig2 |
| Ccdc162 | Acr |
| Bambi | MSTRG.12154 |
| Fosb | Rel |
| MSTRG.28056 | Itga2b |
| Depp1 | Il7r |
| Ezh2 | AC128848.1 |
| Fzd8 | Ank1 |
| Insm1 | Zcchc3 |
| MSTRG.31010 | Icam4 |
| MSTRG.27155 | Mex3b |
| MSTRG.27157 | Cd83 |
| Jun | MSTRG.34461 |
| Cyp1a1 | MSTRG.13607 |
| Lax1 | MSTRG.1996 |
| MSTRG.25644 | MSTRG.27405 |
| Ribc2 | C2cd4b |
| MSTRG.23578 | Pfkfb3 |
| Hist2h3c2 | MSTRG.6019 |
| MSTRG.33277 | NEWGENE_1309147 |
| Derl3 | P2ry6 |
| Gadd45g | Slc30a3 |
| H1f2 | MSTRG.2820 |
| Tbxt | Fam163b |
| Dbh | Per1 |
| MSTRG.23300 | Crem |
| MSTRG.12422 | Chrne |
| Clec18a | Aspa |
| Tcte1 | Sema4c |
| MSTRG.13342 | Gpr171 |
| Fgfbp1 | Socs3 |
| Vcam1 | MSTRG.21393 |
| Krt17 | Sh2d2a |
| AABR07065031.1 | Olr1387 |
| MSTRG.23239 | Ldhc |
| Rasd1 | Hap1 |
| Ccn1 | Cblb |
| Gpr68 | Mbl1 |
| Cldn4 | Lilrb2 |
| Cd180 | Sphk1 |
| MSTRG.29179 | Slc26a9 |
| Ggt1 | Ptgs2 |
| Cx3cr1 | Fam71a |
| Creb5 | Mt2A |
| Hpgd | AABR07021465.1 |
| Sik1 | Ccn2 |
| Trim54 | Zfp60 |
| MSTRG.10665 | MSTRG.32904 |
| RGD1560146 | Traf6 |
| MSTRG.3555 | Zscan12 |
| MSTRG.226 | Csrnp1 |
| Cebpd | Jrkl |
| MSTRG.15341 | Erp27 |
| Itprip | MSTRG.130 |
| Hhex | Cd69 |
| AABR07008066.2 | MSTRG.15920 |
| Zfp36 | MSTRG.20910 |
| Ccdc60 | Il1a |
| Ccdc69 | Il1b |
| Mylk2 | Fam83d |
| MSTRG.21710 | Zfp111 |
| MSTRG.16454 | Syt1 |
| Ier2 | Cebpb |
| MSTRG.16861 | MSTRG.21615 |
| Lpar6 | MSTRG.21610 |
| MSTRG.6689 | Bcl2l11 |
| H3f3a | MSTRG.10970 |
| MSTRG.5403 | Tigit |
| MSTRG.27919 | MSTRG.23698 |
| Atf3 | Eomes |
| Mecp2 | MSTRG.27816 |
| Dnaaf8 | Olr1565 |
| MSTRG.16080 | Ier3 |
| MSTRG.29861 | Irak2 |
| MSTRG.23747 | Il4r |
| Gimap9 | Snx31 |
| Gimap4 | Pim3 |
| Cfap74 | Pim1 |
| Cfap73 | Ifit3 |
| Proser2 | Serpind1 |
| MSTRG.21562 | Mt1 |
| Xrcc2 | MSTRG.5611 |
| MSTRG.25505 | MSTRG.5612 |
| Efhb | Sele |
| Dnai1 | Tnfaip3 |
| Cyp2d2 | MSTRG.28659 |
| Itk | MSTRG.23749 |
| Rrp8 | Lif |
| Capsl | MSTRG.3219 |
| Gvin1 | MSTRG.9514 |
| Dnajb13 | Adora2b |
| Cfap43 | Ubap1l |
| Vmp1 | Ankrd37 |
| Tekt1 | Ier5l |
|  | Apold1 |
|  | Mefv |
|  | Ccl3 |
|  | Ccl4 |
|  | Sox18 |
|  | F3 |
|  | Setmar |
|  | Amotl2 |
|  | Zfp799 |
|  | Trib3 |
|  | Hist1h2af |
|  | Sema7a |
|  | Plat |
|  | Pmaip1 |
|  | Nfil3 |
