# Supplementary material for: Transcriptome profiling and weighted gene co-expression network analysis reveal changes of hub genes and molecular pathways in rat lungs following deep hypothermic circulatory arrest
Source: PLoS One. 2025 Aug 14;20(8):e0328887. doi: 10.1371/journal.pone.0328887 (PMC12352637; doi:10.1371/journal.pone.0328887)
Supplement: S4 Table — MCC: Maximal Clique Centrality. (DOCX) [file pone.0328887.s007.docx]

**S4 Table. The MCC scores of eight hub genes.**

| Rank | Name | MCC Score |
| --- | --- | --- |
| 1 | Fos | 6041 |
| 2 | Egr1 | 6000 |
| 3 | Jun | 5961 |
| 4 | Atf3 | 5670 |
| 5 | Nr4a1 | 5188 |
| 6 | Ccn1 | 5162 |
| 7 | Zfp36 | 5160 |
| 8 | Fosb | 5090 |
